# Collectanea Medica

**Published:** 1814-05

**Authors:** 


### COLLECTANEA MEDICA,

CONSISTING OF

### ANECDOTES, FACTS, EXTRACTS, ILLUSTRATIONS, QUERIES, SUGGESTIONS, &c.

RELATING TO THE

*History or the Art of Medicine, and the Auxiliary Sciences.*

---

*On impeded Deglutition, produced by Organic Derangement of the Œsophagus.* By Dr. HEINEKE.

THE incapacity of introducing food freely and easily by means of the gullet into the stomach, constitutes a disease equally terrible in its effects, and difficult to remove.

The cause of this disorder is either of a transitory or permanent kind; and in the latter case, some organic derangement is generally present, either in the œsophagus or the neighbouring parts; which, contracting, fills up the passage.

---

As

As long as this complaint is transitory in its nature, hopes of its ultimate removal may reasonably be entertained; but these attacks must be attended to at an early period of the malady, and the treatment adapted to the cause of the disorder, otherwise by their frequent recurrence permanent organic disease is produced; to combat which, all the efforts of art prove totally unavailing.

But it occasionally happens that some mechanical impediment being actually present in or near the œsophagus, the introduction of food is effected with facility at some moments, although at others not a morsel of solid, nor one drop of fluid nutriment can be got down The latter case frequently misleads medical men, by offering some grounds for hope, which, however, almost always prove fallacious.

The case which I now offer to the public was of this latter kind. There were intervals during which we were induced to conclude, and not without some reason, that the disease was removed, and yet important, and most probably incurable organic disease was present. The co-operation of an occasional cause must be supposed to have then occasioned the contraction of the œsophagus, which was never so much affected by the organic obstruction as totally to impede the passage of nutriment.

The patient was a man 47 years of age, who had never been afflicted with any considerable malady, and enjoyed tolerable good health. At times, indeed, he was subject to rheumatic cephalalgia, which was mostly very violent.

This state of health continued till nearly a year before his death. About this time several unpleasant events rapidly succeeding each other, produced a very irritable and melancholy state of mind, which, however, did not seem to affect the functions of the body.

But a circumstance happened, which for a considerable time he did not think worthy of his attention. He occasionally felt some difficulty in swallowing, and resistance to the passage of the food into the stomach, which was sometimes rejected, mixed with mucus.

Three months nearly elapsed before he felt any other inconvenience from it, excepting that of sudden inability to swallow, even when eating with the best appetite.

Whole days elapsed, during which he felt nothing of this complaint; on others he could scarcely take any nutriment; and if he swallowed solid food, he could only do it by washing it down with fluids.

He likewise felt an unpleasant pressure upon the chest, about the middle of the sternum, which was increased on swallowing food; he conceived also, that the impediment to deglutition lay thereabouts. He then first had recourse to medical advice.

The only idea which could arise in our minds from the symptoms then present, was that of an organic obstruction of the œsophagus, co-operating with occasional irritation to produce *impeded* deglutition.

Thickening of its coats, their distension, schirrosity of the bursæ mucosæ, or even polypous excrescences, seemed the probable cause of this obstruction. But as there were evident intermissions between these symptoms, the idea of an occasional irritation in the œsophagus from some unknown cause could not be suppressed.

What next struck our attention was the rheumatic diathesis, which seemed to have principally produced the violent attacks of headach. This latter affection had, at this time, almost entirely left that part; but yet occasionally produced pains in the limbs, which, however, were only of transient duration, and constantly varying their seat. The *tinctura antimonii saponacea* was prescribed, and seemed to agree very well with the patient, and to be productive of beneficial effects, as the pressure on the chest was diminished, swallowing rendered less difficult, and the attacks of the disease much less frequent in their occurrence. Encouraged by the above favourable appearances, the use of this remedy was persisted in for several weeks; at last, however, as it failed of producing its beneficial effects, the administration of other medicines was deemed requisite; for the pressure upon the sternum was greatly increased; and the difficulty of swallowing so much augmented, that the patient frequently was unable to take any nutriment for several days; he of course suffered greatly, and evidently lost both flesh and strength.

*Small doses of calomel* with *cicuta* were now had recourse to; blisters were applied to different parts of the body, *oleum cajeputi* with *tincture* of *Spanish flies* and balsamics, were rubbed in upon the chest, &c. which was also covered with a warm plaster.

The use of these remedies was also attended with beneficial consequences. The food passed more freely; the vomiting and pressure of the chest were removed; a more copious perspiration was elicited during the night time; and more violent and continued pains of the extremities came on. We were prevented from joining the tepid bath with these, by the severity of the season. The administration of the mercurial was continued for some short time. The taraxacum and acetated tartar were also occasionally given. The violence of the disorder seemed to abate considerably, as the patient felt much easier; could sometimes eat without impediment, and even return to his usual occupations.

As perseverance in the mercurial course did not seem likely to be of any service, but rather to prove hurtful to the digestion, it was discontinued, and in its stead a mixture was prescribed, composed of extract of taraxacum, and of carduus benedictus, with aqua laurocerasi and aqua aurantiorum; which was employed with apparent benefit for above a month.

The aqua laurocerasi seemed particularly useful; for on increasing its dose, till nearly a drachm was given each time, all symptoms were again alleviated.

This amelioration was, however, not permanent; for even in the beginning of December there were times (and this happened almost daily) when it was not only impossible for him to swallow any nutriment, but it was also observed, that all the food, when it reached the place where the stricture of the œsophagus commenced, was rejected with considerable violence, intermixed with mucus. The spasmodic spasmodic nature of these attacks was so evident, that I resolved upon trying the belladonna, as a remedy particularly operating upon the gullet; the powdered leaves were accordingly given in the dose of a grain three times a-day. No change for the better was however observed; the violence of the symptoms, on the contrary, increased daily.

On account of the favourable appearances which had been perceived during the employment of the calomel, its use was resumed. Six grains were directed to be taken every twenty-four hours, together with camphor, and small doses of opium; and a blister to be applied to the chest.

The decrease and alleviation of the malady consequent upon the employment of these remedies was apparent. No appearance of the disease was observed during several days, the patient ate and drank as when in health; on this account, the same plan was persevered in, without any material alteration during the whole month of December. But the mercurial was then observed to affect the gums, whence it became advisable to omit its use. As rheumatic pains of the joints were moreover observable, and the strength of the patient seemed to be impaired, an infusion of valerian with camphor, *acetite* of *ammonia*, and *extract* of *aconite* was prescribed, and given alternately with similar remedies, such as seneka root, *liquor cornu cervi succinatus, tinctura moschi artificiali,* and opium, &c. according to circumstances. The chest was covered with *emplastrum de galbano*, with the addition of sal. cornu cervi and opium. The above-mentioned favourable appearance of the disease did not only maintain its ground under this plan, but also the pleasing symptoms were daily augmented, so that we had much reason to hope for a final removal of the disease.

This intermission of the disease, however, like the preceding ones, was but of short duration; for by the latter end of January the disorder again increased to such a degree, that the patient was frequently unable to swallow any kind of food for twenty-four hours; he also suffered, during these periods from cramp and unceasing pain in the stomach. A mixture of *assafœtida*, *castor*, and *opium*, given internally, and assisted by the friction of *oleum cajeputi* and opium, upon the region of the stomach, afforded momentary relief from the last-mentioned symptoms, without, however, preventing their recurrence.

My friend, Dr. Albers, was now kind enough to visit this patient with me, and to assist me in the treatment of his complaint.

We determined, as his opinion of the nature of the case exactly coincided with my own, to have again recourse to the use of calomel, in combination with *calcined zinc*, the warm salt-water bath, and the application of blisters to the legs.

A few days after this treatment had been acted upon, the impediment to deglutition was so far removed, that the attacks came on but seldom, and were of short duration; the pains and spasms of the stomach were totally removed. The rheumatic pains, on the other hand, became more frequent, particularly in the left leg, and were often so violent as to deprive him of sleep during the night-time. After each immersion in the bath, copious perspiration came on, which afforded great relief to our patient, who, comparatively speaking, felt very well; the appetite and digestion were good, the evacuations regularly performed, and the pressure upon the chest, which had hitherto remained constant, was now entirely removed. In order to produce more effect upon the skin than could be done by means of the before-mentioned baths, whose beneficial influence upon the local malady, by bringing forth the rheumatic affection to the extremities, was evident, the caustic alkali was mixed with the water of the bath, instead of the table salt, which had been previously employed.

A few days after the employment of these lixivious baths, a powerful re-action came on in the vascular system; considerable febrile action, increased perspiration, and the rheumatic affection of the external parts, particularly of the legs, was induced; and the original disease was but rarely troublesome.

These baths were employed for about a fortnight, and as the mercury again affected the gums and salivary glands, it was omitted, and the employment of the calcined zinc, camphor, and extract of aconite, with the occasional use of opium to mitigate pain, was persisted in. The febrile symptoms now became so violent as to endanger the patient's strength; on this account it was thought advisable to discontinue the use of the bath, and to exhibit internally salep, with elixir of vitriol; costiveness was obviated by gentle laxatives formed of tamarinds, &c.

The fever still continued to gain upon us, notwithstanding the above measures, assuming a synochal character, with a full, hard pulse, great increase of temperature, violent pains in the extremities, without any apparent diminution of strength, or locomotive power.

This state of the disease evidently demanded the use of antiphlogistic, cooling remedies, and the febrile action was after some time considerably lessened by this administration.

During several days our patient found himself remarkably better; slight symptoms of the stricture of the œsophagus were sometimes, though very rarely, remarked.

The scene was, however, suddenly changed, for a violent pain of the stomach, with vomiting of a yellowish, bitter-tasted substance, together with intolerable anxiety and oppression came on. A gentle emetic, consisting of a solution of emetic tartar with castor, evacuated a very large quantity of this greenish yellow substance, which greatly relieved our patient. A mixture composed of citrated kali and tamarinds, produced stools containing the same substance, with like beneficial effect.

A bitter infusion with acid elixir, administered with a view of strengthening the stomach and system, would not agree with the patient; the pressure upon the stomach, and anxiety being increased by it.

The continued inclination to vomit, the bitter taste in the mouth,

 and and foul tongue, evidently shewed the existence of derangement of the biliary system; to remove which became now our principal object. The remedy that appeared most likely to answer this purpose was ipecacuan, and it was accordingly given in the dose of half a grain every hour. We attained our purpose inasmuch as the sickness and bitter taste disappeared, the foulness of the tongue was removed, and some degree of appetite returned. His former sufferings and total *insomnia*, which had continued for upwards of a fortnight, had greatly impaired the strength, and increased the irritability of his system, and affected the brain, so that the patient was restless, uneasy, and delirious; and slight convulsive attacks, with subsultus tendinum, were frequently observed. The pulse was at that time small and weak, though frequent, and indicative of irritation; considerable febrile action came on in the evening. The pain of the limbs, especially of the legs, which had hitherto been pretty violent, was now scarcely perceptible.

A saturated infusion of valerian, with liquor cornu cervi succinatus, and small doses of opium, was now administered; and about twelve hours after their employment, the disease again assumed a favourable turn. A tranquil sleep ensued, which afforded much relief, with evident increase of strength, and a natural, nearly healthy pulse; all the spasmodic symptoms, and the disorder of the gastric system, had totally disappeared. The employment of the valerian, &c. was continued, omitting, however, the opium.

This amelioration was unfortunately but of very short duration; and our patient was again attacked by an unpleasant pressure and fullness about the præcordia and hypochondria, together with great anxiety and difficulty of respiration. These circumstances, and the concomitant constipation, which could not be totally removed even by clysters, occasioned us to prescribe the infusion of valerian with tincture of rhubarb and extract of taraxacum.

This medicine opened the bowels effectually, and afforded relief; but the following night a vomiting of blood came on, the appearance of which was at first black, coagulated, and lumpy; afterwards dark brown thick grumous matter was thrown up, the latter in large quantities. Salep with acid elixir was then administered; the sanguineous vomiting nevertheless continued equally copious and violent for twenty-four hours. The patient's strength decreased rapidly, and death soon closed the scene.

On the day after the patient's death the body was opened and examined. No external preternatural appearance was observed in the extremely emaciated body of the deceased, excepting a swelling about the size of a walnut, at the upper part of the sternum, which, as was afterwards seen, had produced caries, terminating in the destruction of that portion of the bone.

On opening the cavity of the thorax, the lungs, which adhered in all points to the pleura, were of a preternatural colour, and covered with small hard tumours, which were also found in great quantities in its substance. The heart and pericardium were healthy, the latter containing a small quantity of fluid.

On

On inspecting the abdominal cavity, the intestinal canal, pancreas, &c. presented no morbid appearance. The stomach was unusually large, but healthy in its structure. The liver was of an uncommon magnitude, and covered with tumours similar to those which were observed in the lungs. Its substance was exceedingly hard and tough, its colour dark yellow, very dissimilar to that it presents when free from disease. The gall-bladder contained nothing but thick bile; the *ductus choledochus communis*, and other biliary ducts, were healthy and free from obstructions.

In order to ascertain the proximate cause of the impeded deglutition, the stomach, œsophagus, and trachea, were separated and taken out. A considerable mal-conformation of the gullet, which was filled with coagulated blood, was now observed, the middle and upper part being greatly widened; the lower, on the contrary, as remarkably contracted. In order to investigate with greater accuracy the causes of this change, it was carefully separated from the trachea. We now found, that on the spot where the windpipe divides into the two great bronchial branches, there was situated a hard schirrous tumour, about the size of a half-crown, and half an inch thick, which compressed the œsophagus entirely. This alone would have sufficiently accounted for the difficulty in deglutition; but with a view to be certain whether the internal texture of the gullet had been changed, it was examined, when an appearance was observed of a very singular kind. The internal surface was of regular and healthful structure and colour, except on the superior portion of its tunica villosa; the remaining portion, to within the distance of two fingers' breadth from the upper orifice of the stomach, was covered with a red, fleshy, and in some places schirrous mass, which so considerably filled up, and narrowed the calibre of the œsophagus, that a small quill could with difficulty be passed through it. This was much harder and thicker towards the inferior extremity of the œsophagus, where it projected like a thick fleshy tongue, nearly closing the canal. The mass was hardest where the above-mentioned external schirrous induration between the œsophagus and trachea was situate. For nearly the length of two inches below this preternatural conformation, the œsophagus regained its healthy appearance, and descended in that manner into the cardia.

---

*Case of Suppression of Urine from Stricture, succeeded by Gangrene of the Arm;* by S. MARSHALL, Surgeon, Leeds.—J. E., an old worn-out quarter-master, while at sea, complained, on the 15th of May, of an inability to pass the bougie, (which he had been long in the habit of doing himself, on account of several strictures in the urethra,) in order to empty the bladder. I attempted, for some time, to introduce bougies of various sizes and forms, as well as catheters, but all to no purpose. As the belly was now become tense, from the fulness of the bladder, and the patient was in severe pain, I gave directions for the warm-bath to be prepared; but as this was likely, from the gallery-fire having been put out, to occupy a considerable a considerable portion of time, he was put to bed, and ordered a draught of thirty drops of laudanum, and in two hours this dose was repeated, soon after which he fell asleep. I now began to revolve in my mind which would be the most eligible mode, on the failure of the warm-bath and other means, with the increased urgency of the symptoms, of puncturing the bladder. The great height, however, of one of the strictures, and the probable disease of the prostate gland, rendered the very simple and ingenious method recommended by Mr. Astley Cooper impracticable, and I determined on that by the rectum. But, happily, during the sleep of the patient, the bladder was in part emptied involuntarily; and, on waking, he was enabled to pass the bougie as well as ever, and complete the evacuation. Thus far the issue of the case was favourable; but the poor fellow only escaped this operation, by and bye to undergo one, in his present state of debility and emaciation, rather more formidable. He passed a tolerably easy night, discharging a little urine frequently; but in the morning, on wakening, found the use of the right arm entirely gone. On being called to him, I found the whole cubit benumbed, cold, and stiff, and not the least pulsation of the brachial artery to be perceived in any of its course; nor was I more successful in feeling that of the subclavian, in passing over the first rib. The limb, above the elbow also, was robbed, in a great measure, of its vital heat, though not so completely as the lower portion. The hand and fingers, in particular, were completely dead. The pulse of the opposite wrist was at the same time very feeble, and his general debility very great. This I immediately began to combat with a more generous and fresh diet, wine, bark, and opiates; ordering the limb affected to be constantly stuped with warm fomentations, alternated with a strong solution of camphor in rectified spirit; but the vital warmth was gone for ever; and in spite of the unremitting exhibition of a variety of the most powerful stimuli, external and internal, the fore-arm continued entirely devoid of sense. On the third day, the fingers became livid, and soon after, by degrees, the whole hand was also discoloured. At this time he complained of soreness from the elbow to the wrist, soon after which the latter part became also livid. On the 30th of May, (having waited some time for the line of separation forming between the mortified and sound parts, and this having now manifested itself in numerous vesicles, and an irregular kind of sulcus, a little below the elbow,) I suggested to the old man the propriety of amputation; but must confess, that my motive for this step was rather the removal of a nuisance, which, from its intolerable stench, had for some days distressed the whole crew, than any expectation of seeing my tottering patient recover after it; indeed, I had, for some time, been prepared for his sinking under his excessive debility. I, however, seized a favourable opportunity, when his vital powers were somewhat recruited, and performed the operation, with the assistance of my mate, and the purser; making the first incision a little above the elbow, and finishing it, with the loss of but about two drachms of blood. The result turned out far beyond beyond my expectation. On the fourth day the wound was in some measure healed by the first intention, and in a month was firmly and completely cicatrized; and the old man, although in rough squally weather, and near the close of a long voyage, was in better health than he had been for a long time, insomuch that he was able to resume his post in directing the steersman.—*Edinburgh Med. and Surg. Journal.*

---

*Biographical Memoir of* M. CABANIS, *by* M. GINGUENÉ.—Peter John George Cabanis, distinguished by his talents as a physician, a philosopher, and a man of letters, was the son of John Baptist Cabanis, who had been brought up to the profession of the law, but who had latterly devoted himself to agricultural pursuits, and was born at Conac, in 1757. Placed at the age of seven under the care of two worthy clergymen, who were brothers, and of whom the one had resigned his living to the other, "he already shewed indications of talent, and evinced, in particular, a degree of steadiness and perseverance which gave reason to hope, that, if he pursued a proper path, he might have some success in the world."* At the age of ten, he was removed to the college of Brive, then under the superintendence of doctomists. "Even before he quitted the lower classes, it was easy to perceive that harsh treatment did not succeed with him; and some ill-timed severities began to give a degree of obstinacy to his character, which adhered to it for a long time." In the second class, however, he took a different turn: under the tuition of a master, as good and amiable as he was well informed, he became docile and studious from motives of affection; acquiring a lively taste for literature, and a sort of passion for the great masters of poetry and eloquence which were put into his hands. His year of rhetoric did not pass so well. Disgusted with the severe treatment which he received from one of the directors of the seminary, he was carried along by the violence of his disposition; his stubbornness returned with twofold force; he was guilty of several provocations towards his masters; he even allowed himself to be accused of a crime which he had never committed; at length he exhausted the patience of his teachers, and was sent back to his father. But he found the harsh usage he met with under the paternal roof more insupportable even than that from which he had just escaped. "His mind revolted from it, and his temper became still more soured, till at last he attended to nothing. At the end of a year, however, his father saw that it would be necessary to adopt some other method with him than that of severity: accordingly, he took him to Paris; and soon perceiving that his superintendence had no beneficial influence on his son, he left him to himself, in the midst of that great city, at the age of fourteen. The expedient was perilous, but it was attended with complete success. Cabanis no

* This, and some of the following passages marked with inverted commas, are taken from a sketch of his life, written by Cabanis himself, which is preserved in his family.

sooner sooner felt himself free from the yoke, which all his faculties had been strained to shake off, than the taste for study revived in him with a species of furor. Little assiduous in his attendance on the professors of logic and physics, he read Locke, and followed a course of lectures by Brisson, while in his leisure hours he renewed his acquaintance with the different branches of his early education. Two years passed with him like a single day, in the company of the Greek, Latin, and French classics, and in the society of certain of his fellow-students, in whom the same taste for letters was combined with amiable manners." While thus engaged, he unexpectedly received a letter from his father, recalling him to the country, and almost at the same time had an offer made to him of the place of secretary to a Polish nobleman. "Having to decide between the choice of a distant journey, which would derange his studies, but left him the hope of resuming them, and a state of complete retirement in the bosom of his family, where the developement of his talents would have been checked for ever, he did not hesitate long: at the age of sixteen he resigned himself into foreign hands, and went by sea to visit a country which had been represented to him as half savage." This was in 1773, during the Diet, in which it was attempted to obtain the consent of the Poles to the first partition of their country. The expedients of terror and corruption which were employed on the occasion, presented a sad spectacle to him, "which inspired him with an early contempt for mankind, and a melancholy which his natural good sense had great difficulty in conquering." After an absence of two years, and at the age of eighteen, he returned to Paris. Turgot, the friend of his father, was at that time minister of finance. He was presented to him, and kindly received by him; and was promised an appointment suited to his talents and his taste, when an intrigue at court displaced the minister. A premature experience, little calculated to give him a relish for the world, and the acquirement of the German language, were the only fruits of his travels. It was requisite to make up for this loss of time, which he resolved to do without delay; and his father, having become more sensible of the necessity of seconding his endeavours, assured him the means of living for two or three years more. Cabanis asked for nothing further. He was united by friendship with the poet Roucher, who was then in great celebrity. This connection revived his taste for poetry; and the French Academy having offered a prize for a fragment of a translation of Homer, Cabanis not only aspired to the reward, but determined to undertake an entire translation of the Iliad. The two pieces which he transmitted to the Academy were not so much as noticed by that body, but several men of taste judged favourably of them; and the passages which were inserted soon afterwards in the notes to the poem of *Les Mois*, obtained universal approbation. The introduction to society which these performances procured him, the invitations to recite his compositions, and the applause of certain persons who were then the dispensers of fame, did not long fascinate him. The blank of this mode of existence increased his melancholy; an excessive application to study produced a considerable derangement of his health; he had no certain prospects before him; and his father was urging him to fix on some useful profession. At last he decided to follow that of medicine, "of which the varied studies offered ample scope for the activity of his mind, and of which the practice requires continual bodily exercise, now become a matter of indispensable necessity to him. His bad health had even some influence in this determination; and he was still further strengthened in it by Dr. Dubreil, to whose advice he had had recourse, and who offered himself as his guide in the new career into which he was about to enter." Cabanis laboured six years under this able master; following him to the bedside of his patients both at the hospital and in his private practice, consulting him about every thing which he saw, and about every thing which he read, and allowing himself to be diverted from his studies by nothing but the cares which his health required. These rendered a residence in the country necessary, and the profession which he had embraced, and was ardently devoted to, required it to be in the neighbourhood of Paris: he made choice of Auteuil. It was there that he became acquainted with the widow of Helvetius, "with that excellent and respectable woman, who since has always treated him as a mother, and whom he has loved as a tender and affectionate son. It was in the society of Madame Helvetius that he continued to cultivate the acquaintance of Turgot; that he became known to d'Holbach, Franklin, and Jefferson; and acquired the friendship of Condillac and Thomas. It was at the house of Turgot and of d'Holbach that he lived many years on a footing of familiarity with Diderot and d'Alembert, and other distinguished men of letters. On the occasion of Voltaire's last visit to Paris, he was introduced to him by Turgot, and read to him some passages of his translation of Homer. The old man, though fatigued and infirm, appeared to listen to them with interest; he praised them much, but it must be confessed, that it was almost always at the expense of the original." Cabanis had for a long time abandoned the prosecution of this undertaking. Absorbed in the studies and labours of his profession, he had entirely renounced the belles lettres; "and his renouncement was so complete and decided, that he spent several years without permitting himself to read a passage of Homer, Virgil, or Racine." He bid adieu to poetry by his *Serment d'un Medecin*, which is a free imitation of the Oath of Hippocrates. This little piece, which was composed in 1783, is valuable, as affording evidence of what his sentiments then were. He became confirmed in them more and more in proportion as the revolution drew near; and when it had broken forth, he shewed himself as much attached to the principles on which it was founded, as he was hostile to the atrocities by which it was sullied. In 1789, he published his *Observations on Hospitals*, before he had been appointed commissioner of those of Paris. Congeniality of sentiment, and mutual connections, had rendered him intimate with Mirabeau. The genius of that extraordinary man, of whom so much good and so much evil may be said, levied contributions from the pens of several men of talent, who were content to resign to him their ideas and their performances, in the persuasion that he would only use them for the general good. Cabanis, having connected himself with him, considered it his duty to join this disinterested association; and it was to him that Mirabeau was indebted for the *Essay on Public Instruction*, which was found among his papers after his death, and published by Cabanis himself in 1791. In his last illness, Mirabeau would have no other medical assistance but his; he died, in a manner, in his arms; and Cabanis shortly after published the *Journal of his Illness and Death*. This attachment, and the accusations of various descriptions which were raised against the object of it, exposed Cabanis himself to much unjust reproach. It is easy to perceive, that the fascination of great talents, the seducing influence of amiable qualities, the admiration with which ideas replete with grandeur and dignity could not but inspire him—it is easy to perceive, that all these circumstances had produced an illusion on his senses, which nothing could dispel, and that the purity of his own mind had rendered him incredulous as to every thing which could sully the memory of the man who had died his friend. Another intimacy which he formed, which was still more strict, and which does not demand the same apology, was that with Condorcet. "Previously to the revolution, he had met him, at Turgot's, at Franklin's, and at the houses of some others of their common friends. Relations of a more intimate nature tended afterwards to confirm the friendship with which personal esteem, and admiration of his great acquirements, had originally inspired him. The misconduct of the revolutionary government, and the horrible persecution to which Condorcet was subjected soon after the 31st of May, strengthened their friendship still more; but all endeavours to save the latter from his unhappy destiny were vain; and, at the fatal catastrophe, Cabanis had only the consolation of receiving the last compositions of his unfortunate friend, and collecting his last wishes in regard to his wife and child. It was shortly after his death, that Cabanis married his sister-in-law, Charlotte Grouchy, sister of the general of that name, and of Sophia Grouchy, the widow of Condorcet." To this union he owed the happiness and the consolation of the remainder of his life. In the third year of the republic, after the reign of terror, when the Central Schools were established, Cabanis was named Professor of Hygiene to the School of Paris; in the year IV, he was elected Member of the Class of Arts and Sciences of the National Institute; in the year V, he was appointed Professor of Clinical Medicine at the Medical School of Paris; and in the following year he was chosen as a representative of the people in the Council of Five Hundred; he continued in this situation till the revolution of the 18th of Brumaire in the year VIII, soon after which he was named one of the members of the Conservative Senate. For some years, however, his health had been becoming more and more infirm; his sensibility, which was naturally so quick and lively, had been increased by continued study and attention to business. In the spring of 1807, after a gentle sleep, he was seized with apoplexy. Fortunately, M. Richerand was just coming into the house at the time, and by his prompt assistance, the disorder was soon stopt in its course; but after this attack, Cabanis was obliged to forsake his studies, and to confine himself more than ever to retirement in the midst of his family.

The vicinity of Paris exposing him to frequent visits, he left Auteuil, and went to reside at the country seat of his father-in-law, M. de Grouchy, twelve leagues from Paris, near the small town of Meulan. He passed all the summer there. The exercises of riding and sporting seemed to be of service to him; he returned by degrees to his books; and had some hopes that he would be able to revise and finish his translation of Homer. In the exercise of his benevolence, however, he found the most agreeable employment of his time; people came to him from all quarters to consult him for poor patients: sometimes he went to visit them himself; and when unable to do this, he gave them advice and pecuniary assistance; being seconded in these acts of beneficence by a nephew, the admirer of his talents, and the imitator of his virtues.

Towards the close of the year, instead of returning to Auteuil, he removed only a little nearer to Meulan, taking a house near the little hamlet of Rueil. He there passed the winter, engaged in the same pursuits, but experiencing more frequent attacks of his complaint, which increased his weakness, and warned him of his approaching dissolution. He would speak of it, and always with perfect composure, and with an affecting tone of melancholy. At length, on the 5th of May, 1808, on returning from a walk, during which he had been engaged in a most affectionate conversation with his wife, he went quietly to bed, slept a few hours, and about one o'clock of the following morning had a fresh attack, which carried him off, notwithstanding the most speedy assistance that could be procured. Thus died, at the age of about fifty-two, one of those men who have united in the highest degree eminent intellectual talents with the virtues of the soul, with nobleness of character, and with the most exquisite goodness of heart. This last-mentioned quality, which governed all his conduct, also pervades the whole of his writings. There is not one of them which does not appear to be dictated by an ardent philanthropy, and by a desire to render his fellow-men better and happier. The only work of his which can be considered as purely literary, is entitled *Melanges de Literature Allemande ou Choix de Traductions de l'Allemand, &c.* 8vo. Paris, An V, (1797.) It is dedicated to Madame Helvetius, and contains nine pieces, of which six are translated from the German of Meissner; a dramatic work by Goethe, entitled *Stella;* a translation of Gray's *Elegy in a Country Church-yard;* and *The Death of Adonis,* from the Greek of Bion. Shortly after he published a work on medical philosophy, in which he examines *The degree of Certainty that is to be ascribed to Medicine,* 8vo. Paris, 1797; reprinted in 1802, with a new edition of his *Observations on Hospitals,* and the *Journal of Mirabeau's Illness.* On this work work we find some remarks by a physician of considerable talent and repute. "The question concerning the certainty of medicine," says M. Pariset, "involves another, viz. Whether there is such a thing as medicine? On this question Cabanis has collected all the most specious arguments which the enemies of the science have ever urged against it; and after having displayed them in all their force, he successfully refutes them, and confounds his opponents with their own weapons. As medicine is nothing more than the art of operating on the human frame in a certain manner, and with certain views, and as every thing in nature has this agency, it is clear, that if there can be any doubts on the subject, it is not so much, whether medicine really exists, as whether it would be possible for it not to exist. With regard to the original question, viz. Whether it be possible to reduce this operation on the human system to certain fixed and invariable rules, and to produce at will such and such an effect, it is evident that this question is much more difficult of solution than the former, and that the certainty of which we are in quest, resolves itself into a greater or less degree of probability, or a greater or less approximation to demonstrative truth; in which respect the science of medicine resembles the other sciences by means of which man is influenced; as, for instance, the science of ethics, and its two principal subdivisions, legislation and politics. It cannot be denied, however, that this dissertation of Cabanis bears the stamp of a mind accustomed to handle the most delicate problems, and to trace the solution from amidst all the elements that obscure it."* Under the title of *Coup d'Œil sur les Révolutions et sur la Réforme de la Médecine*, Cabanis produced a work in which the various doctrines of the great men, who have, at different periods, influenced the progress of science, are explained and illustrated, with an analytical talent, and by such judicious comments, as render this work itself an instrument of reform and improvement. He has also left, 1. A treatise of no great extent, but highly esteemed by professional men, entitled *Observations sur les Affections Catarrhales en general, et particulierement sur celles qui sont connues sons le nom de Rheume de Cerveau, et Rheume de Poitrine*, 8vo. Paris, 1807. 2. Several essays on subjects of philosophy, politics, and other sciences, in different literary journals, and among others in the *Magazin Encyclopedique, a Dissertation on the Punishment of the Guillotine*, controverting the opinion of Sœmmering and Sue, who regard this mode of punishment as extremely painful, and even think that the pain is felt after death. 3. In the political journals, especially in the Moniteur, several *Speeches* delivered in the Tribunate and in the Council of Five Hundred. But the great work of Cabanis, and the most solid monument of his fame, is that in which he explains the *Relations of the Physical and the Moral Constitution of Man*. Six out of the twelve memoirs of which it consists, were

---

* Notice historique et litteraire sur Cabanis, lue à l'Athénée de Paris.

originally originally printed in the two first volumes of the *Recueil de l'Institut National, Classe des Sciences Morales et Politiques;* they were republished with the six last, in 2 vols. 8vo. Paris, 1802; and in the following year a second edition appeared, with corrections and additions by the author, accompanied with a Systematic Analysis and Recapitulation, by the Senator M. Destutt Tracy, and with an Alphabetical Index of the Authors and Subjects, by M. Sue, Professor of the School of Medicine at Paris. This work occasioned some animadversions, from which the formal declarations of the author in different parts of his work ought to have saved him. "Some persons," he observes in the preface, "have appeared, as I have been told, to apprehend, that this work was designed, or calculated, to subvert certain doctrines, and to establish others in their stead, with respect to the nature of *first causes;* but that is certainly not the case, and with a little reflection and candour, no one can seriously believe it. The reader must often remark, in the course of the work, that we consider these causes as beyond the sphere of our research, and entirely inscrutable by the faculties of investigation with which man has been endowed along with life. We here make this explicit avowal of our sentiments on this point; and, if it were necessary to say any thing further with regard to questions which have never been discussed with impunity, it would be extremely easy to prove, that they cannot become either the subject of examination, or even a subject of doubt, and that the due employment of our reasoning powers leads us only to a state of insuperable ignorance with regard to them. We shall therefore resign to more confident, or if they chuse rather to be termed more enlightened minds, the task of investigating, by paths which we consider as shut to us, the nature of the principle which animates living bodies, &c." Assuredly, the spirit of true philosophy never spoke with greater circumspection, modesty, and wisdom. But whatever may be the nature of the living principle, it acts, and operates within us. In what manner does it so act? What part of our frame is the principal seat of its action? What are its operations? These are the questions which Cabanis endeavours to determine. Locke was the first who cleared the way for this inquiry, by clearly explaining and establishing the old fundamental axiom, *that all our ideas are derived from our senses, or are the result of our sensations.* Condillac enlarged and improved the doctrines of Locke. His disciples have improved upon them still further; and some of them have corrected, in several material points, his view of the processes of the understanding: but there was still wanting a more complete examination and exposition, than had been given by Condillac and his school, of the functions and the developement of the organs which are subservient to the formation of ideas. *All our ideas are derived from our senses.* Granted: but in what manner are they so derived? and how do our sensations produce ideas? These questions, it is obvious, belong entirely to the province of physiology; and it is by collecting all the facts which the progress of this science in the present age has furnished, that our author endeavours to determine termine them. In the first memoir, he offers some general reflections on the study of man, and on the connection between his physical organization and his moral and intellectual faculties; in the second and third, he gives the physiological history of his sensations, following in some measure the course which they take, and the changes which they undergo, from the extremities of the nerves which receive the first impressions of external objects, to the brain, where they originate, and where they all terminate: to this organ they convey all these impressions; and it is there that they are converted into ideas. The brain is therefore the common centre where this operation is effected, and whence all exertion of thought proceeds. We may easily imagine, then, by what a variety of causes its actions may be influenced; some of them being inherent in the individual, and inseparable from it; others being extraneous and accidental. In the six following memoirs, Cabanis considers the manner in which the formation of ideas and moral habits is influenced by age, by sex, by the temperaments, by disease, by regimen, and by climate. The tenth memoir treats of animal life, and the first determinations of sensibility, of instinct, of sympathy, of sleep, and of delirium. Having fully examined all the circumstances which may affect our actions and moral habits, he proceeds, in the eleventh memoir, to consider the reciprocal influence, or the re-action of our moral on our physical constitution. Regarding always, as he does through the whole of his work, the brain, as the organ which must, according to the laws of the animal economy, be exerting the most constant, the most general, and the most powerful action, he draws the conclusion, that this evident re-action of the moral on the physical constitution, is nothing else than the influence of the cerebral system, as the organ of thought and volition, on the other organs, of which it is capable of exciting, suspending, or even deranging all the functions, by means of its sympathetic action. Lastly, in the twelfth memoir, he discusses the subject of acquired temperaments: this may be considered as a sort of supplement to the fourth, which treats of the moral influence of the temperaments, and in which he had only considered the natural or congenital temperaments, or those to which individuals are predisposed from their birth. Under the term of *acquired temperaments*, he meant those temperaments which are formed in different individuals, by a long continuance of those accidental impressions to which they are exposed, such for example as those that originate from disease, from climate, from regimen, and from particular employments of the body and mind. Our limits forbid us to extend this meagre analysis of the work before us; but, imperfect as it is, it may convey some idea of the grandeur, importance, and novelty, of the questions and problems, which the author has endeavoured to investigate. He has proceeded in the execution of his task, with a degree of method which aids the understanding, and with such candour and ingenuousness as ought to have secured him from the attacks which have been made upon him. He was apprised of these attacks, and he has condescended to reply to them in the second edition of his book.

He

He had refrained, as we have already seen, from the consideration of the great and delicate question of *first causes;* but he afterwards ventured on the discussion, displaying in it a great superiority of talent, of reason, candour, and information. The results to which he is led, prove that his private sentiments were very different from what they were by some people imagined to be. This composition deserves to be ranked among the finest compositions of abstruse philosophy of which our language can boast. His family is in possession of another valuable though unfinished work, viz. the translation of more than half the Iliad. These fragments, and some others of a various nature, which were left by Cabanis, could not but be favourably received by the public.

